# Wide-awake vs. regional anesthesia for carpal tunnel release: a prospective study on pain, grip strength, and sleep quality

**DOI:** 10.1186/s12893-025-03275-z

**Published:** 2025-11-04

**Authors:** Alkan Bayrak, Mustafa Yalın, Murat Tıngır, Tevfik Çatal, Vedat Öztürk, Ali Can Koluman

**Affiliations:** 1 Department of Orthopedics and Traumatology, Istinye University Gaziosmanpaşa Medical Park Hospital, Istanbul, Turkey; 2 Department of Orthopedics and Traumatology, Yalova University Faculty of Medicine, Yalova Training and Research Hospital, Yalova, Turkey; 3 Department of Orthopedics and Traumatology, TC Sağlık Bakanlığı Of Devlet Hastanesi, Trabzon, Turkey; 4 Department of Orthopedics and Traumatology, TC Sağlık Bakanlığı Kahramanmaraş Necip Fazıl Şehir Hastanesi, Kahramanmaras, Turkey; 5 Department of Orthopedics and Traumatology, Bakırköy Dr. Sadi Konuk Eğitim ve Araştırma Hastanesi, Istanbul, Turkey

**Keywords:** Carpal tunnel syndrome, WALANT, Axillary brachial plexus block, Pain management, Functional recovery

## Abstract

**Background:**

Carpal tunnel release (CTR) is a common procedure for carpal tunnel syndrome (CTS), with WALANT (Wide-Awake Local Anesthesia No Tourniquet) and Axillary Brachial Plexus Block (ABPB) as two primary anesthesia options. This study compares their effects on pain, functional recovery, grip strength, and sleep quality.

**Methods:**

A prospective observational study with quasi-randomization was conducted on 62 patients undergoing CTR under WALANT or ABPB. Outcomes included operative time, Visual Analog Scale (VAS) for pain, grip strength, and Pittsburgh Sleep Quality Index (PSQI). Patients were followed up at 1 week, 3 weeks, and 3 months postoperatively.

**Results:**

WALANT significantly reduced operative time compared to ABPB (mean: 20.8 ± 4.1 min vs. 41.5 ± 9.4 min; mean difference: 20.7 min; 95% CI: 16.3–25.1; *p* = 0.001; d = 2.45). It was also associated with significantly lower pain scores at both 3 weeks and 3 months. At 3 weeks, VAS scores were 0.7 ± 0.8 (WALANT) vs. 2.5 ± 2.1 (ABPB) (mean difference: 1.8; 95% CI: 0.6–2.9; *p* = 0.003; d = 1.00), and at 3 months, 0.3 ± 0.4 vs. 2.3 ± 1.4, respectively (mean difference: 2.0; 95% CI: 1.1–2.9; *p* = 0.003; d = 1.15). Early postoperative pain scores (1st hour and 1st day) were also lower in the WALANT group; however, these differences did not reach statistical significance. Grip strength recovery at 3 months showed no significant difference between groups in both thumb pinch and finger pinch measurements (*p* > 0.05). Similarly, sleep quality improvements assessed by PSQI did not significantly differ between groups at 3 months (WALANT: 5.7 ± 1.6 vs. ABPB: 5.9 ± 1.3; *p* = 0.868).

**Conclusion:**

WALANT is a time-efficient and effective anesthesia method for CTR, offering prolonged pain relief without compromising functional recovery, grip strength, or sleep quality.

## Introduction

Carpal tunnel syndrome (CTS) is the most prevalent peripheral nerve entrapment disorder globally, impacting millions of individuals annually [1–3]. Carpal tunnel release (CTR) as a surgical intervention has shown a rise in both popularity and prevalence [4, 5]. This tendency has prompted substantial study to contextualise the settings and variables that enhance CTR. Wide-awake, local-anesthesia, no-tourniquet (WALANT) procedure has become a viable, safe, and cost-efficient alternative for several surgical interventions affecting the hand, including CTR [6]. In a 2020 study of American hand surgeons, about 80% indicated they had conducted WALANT procedures during their careers, while more than 60% were actively employing WALANT in their clinical settings [7]. Since then, its recognition and utility have continued to expand, particularly during the current COVID-19 pandemic, when its practicality in outpatient procedures and superior infectious safety profile, due to the elimination of aerosol-generating anaesthesia, were favoured [8, 9].

Monitored Anaesthesia Care (MAC) for hand and forearm surgery often includes Axillary Brachial Plexus Block (ABPB) to manage pain and maintain limb immobility, while sedation is administered to alleviate patient anxiety [10]. Current recommendations advocate for the utilisation of ultrasound-guided ABPB, since it enhances the efficacy of the block and reduces the incidence of complications [11]. CTR is the most often executed hand operation; however, it continues to be a topic of contention among the hand surgery field. The kind of anaesthesia, surgical method, and site of CTR differ according to surgeons’ preferences, despite minimal data indicating similarity in both operation and anaesthesia type [12].

While the choice of anesthesia in CTR is often dictated by surgeon preference, its potential impact on recovery parameters such as pain, sleep quality, and grip strength remains underexplored. Therefore, we selected the 3-month mark for outcome assessment as it signifies a crucial phase where temporary surgical effects decrease and long-term functional recovery becomes stable. Sleep disturbances in CTS are anticipated to improve following decompression; however, the degree and pace of recovery may be affected by the management of postoperative pain, which differs according to the type of anaesthesia used. WALANT has demonstrated the ability to decrease postoperative pain, potentially facilitating a quicker return to normal sleep patterns. In a similar vein, while the decline in grip strength is mainly attributed to the release of the transverse carpal ligament (TCL), early mobilisation could contribute to the pace of recovery. WALANT facilitates immediate hand movement during surgery, which may enhance the speed of functional rehabilitation in contrast to ABPB, where there is a period of temporary limb immobility. We hypothesized that WALANT would offer enhanced postoperative pain management in both the early and mid-term phases, resulting in a quicker enhancement in sleep quality compared to ABPB. Furthermore, we anticipated that early mobilisation with WALANT would improve grip strength healing at three months following the operation. This study aims to assess the influence of anaesthesia type on mid-term pain levels, quality of sleep, and grip strength after CTR.

## Methods

### Study design

After approval by the local ethics committee, this prospective observational study with quasi-randomization was conducted and did not require clinical trial registration (Clinical trial number: not applicable). We identified symptomatic patients with hand numbness and sensory loss who were clinically diagnosed with CTS and confirmed by electromyographic (EMG) findings. A signed informed consent was obtained from all participants before recruitment. Prospectively and consecutively, all patients who underwent CTR were invited to participate. To allocate patients to either the WALANT or ABPB group, a date-based randomisation method was implemented. Patients scheduled for surgery on odd-numbered calendar days received WALANT, while those scheduled on even-numbered days underwent ABPB. This method was chosen to balance methodological rigor with logistical feasibility. Unlike computer-generated randomisation, which may disrupt operating room scheduling and resource planning, the date-based approach allowed for consistent perioperative preparation, optimal use of anesthesia staff, and adherence to institutional workflow. This systematic methodology provided that patient choice did not affect anaesthesia selection, hence minimising selection bias. Due to the inherent differences between the anaesthetic techniques (e.g., tourniquet use, sedation level, intraoperative communication), full blinding of patients and surgeons was not feasible. However, postoperative outcome evaluations were conducted by independent assessors who were blinded to the anaesthesia type, and not involved in the surgical procedure or perioperative care. This approach ensured a reasonable level of assessor blinding, particularly for subjective outcomes such as pain and sleep quality. Patients received standardised rehabilitation and pain management recommendations to reduce potential variations in recovery experiences attributable to knowledge of anaesthesia type. The complete blinding of patients was not achievable due to the unique characteristics of the anaesthesia techniques employed. However, to minimise bias, patients were not explicitly informed about the study hypothesis or any potential differences in outcomes related to the type of anaesthesia used. All participants and investigators followed a standardised data collection protocol to ensure objectivity in evaluations.

### Patient selection

Eligible patients aged 18 years and older were those with a clinical and EMG-confirmed diagnosis of CTS. The following criteria were used to exclude patients: age less than 18, history of CTR surgery on the hand that was going to be operated on, or CTS due to acute or traumatic conditions. Both endoscopic CTR and concurrent operations (such as CTR with trigger finger release) were also excluded. Also excluded from the research were patients who needed general anaesthesia, had pathological masses on their wrists, had been in for wrist surgery before, had contractures in their hands or wrists, had brachial plexus injuries, or had median nerve injuries. In addition, patients with systemic disorders known to affect peripheral nerve function, such as diabetes mellitus, hypothyroidism, or polyneuropathy, were excluded. Although these criteria aimed to minimise confounding, certain potential unmeasured variables were not systematically recorded, such as hand dominance, occupational status, and baseline sleep disturbances. However, the study population comprised a relatively homogeneous group in terms of socioeconomic background and access to healthcare services. In addition, no patients had documented diagnoses of chronic sleep disorders or psychiatric illnesses. This limitation has been acknowledged and discussed in the relevant section.

### Surgical procedure and follow-up

Prior to preparation, the anaesthetic injections were administered into the inter-thenar region along the scheduled incision in a subcutaneous plane. The injection comprised 10 cc of 1% lidocaine combined with 1:100,000 epinephrine and 1 cc of 8.4% sodium bicarbonate. (WALANT consortium). A mini-open CTR was conducted 10–15 min following the injection. After appropriate preparations and draping, a longitudinal incision measuring 3–4 cm was made from the volar aspect of the wrist over the palmar aponeurosis and transverse carpal ligament (TCL). CTR was performed, which involved cutting the transverse carpal ligament to relieve pressure on the median nerve. Under the guidance of an ultrasound probe and a nerve stimulator, the ABPB group had upper-limb anaesthesia via an axillary block with a mixture of 0.5% bupivacaine (20 ml) and 2% lidocaine (10 ml). In the ABPB group, the tourniquet was applied on the upper arm to minimize blood loss, then deflated after the surgery. A retractor was employed to generate local pressure for the duration necessary to finish the release in cases when a bleeder was detected and impacted visualisation. The skin closure was the sole procedure that effectively controlled the bleeding. The patients were all closely watched using noninvasive blood pressure monitors, electrocardiograms, and pulse oximeters. In order to minimize confounding variability, all surgeries were conducted by trained hand surgeons using a standardized technique. In the WALANT group, local anesthetic injection was administered by the operating surgeon in the operating room. In the ABPB group, the block was performed by a board-certified anesthesiologist under ultrasound guidance prior to surgical preparation. Clinical outcomes (functional recovery and complications) were assessed one hour, eight hours, one day, three weeks, and three months following surgery. Patients were followed carefully for compliance with the planned follow-up schedule, and those with no data were excluded from the final analysis.

### Outcome assessments

The primary outcome of the study was postoperative pain at 3 months (Visual Analogue Scale -VAS), with secondary outcomes including postoperative grip strength and postoperative sleep quality (Pittsburgh Sleep Quality Index -PSQI). Blinding was not feasible for early postoperative outcomes (1 h, 8 h, and 24 h), as patients could infer anesthesia type based on intraoperative experience. These assessments were therefore subject to potential detection bias. However, the primary outcome (VAS at 3 months) was evaluated by an independent assessor blinded to group allocation. The main included outcomes were operative time (from operating room entry to exit), pain, and functional recovery. Operative time was defined as the total duration from the moment the patient entered the operating room to the time they exited after surgery. This measurement included all intraoperative activities, such as anesthesia administration (either ABPB or WALANT), patient positioning, sterile preparation, and the surgical procedure itself. Both anesthesia techniques were performed within the OR, ensuring that no additional time advantages were conferred by external preoperative preparation. By incorporating the full intraoperative period, this approach allowed for a more comprehensive comparison of patient flow and efficiency between WALANT and ABPB. Pain was assessed with the VAS before and several times after surgery (1 h, 8 h, 1 day, 3 weeks, and 3 months). Functional recovery was evaluated by measuring the grip and pinch strength obtained before the operation and at 3 months after the operation.

The secondary outcomes were patient-reported sleep quality assessed by the PSQI, which evaluates the different components of sleep disturbance. The Boston Symptom Severity Scale (SSS) and the Boston Functional Disability Scale (FDS) were used to assess symptom severity and functional disability. Improvements were evaluated by comparing the differences in these scores between time marks (preoperative vs. postoperative 3 months). Additionally, the severity of carpal tunnel syndrome based on EMG results was classified into subtypes (Type 2 and Type 3), and the TCL thickness was evaluated through ultrasonography to analyze structural changes in the wrist.

### Statistics

Statistical analyses were performed using IBM SPSS version 26 (Chicago, IL, USA). Descriptive statistics (mean, standard deviation, minimum, maximum, median) were computed for all variables. The distribution of continuous data was assessed using the Shapiro–Wilk test and visual histogram inspection. For between-group comparisons, the independent samples t-test was used for normally distributed variables, and the Mann–Whitney U test for non-normally distributed variables. Within-group differences over time were assessed using the Wilcoxon signed-rank test. The primary outcome of this study was the difference in VAS pain scores at 3 months postoperatively. Although an a priori power analysis was not feasible due to constraints in patient enrollment during the study period, we conducted a post hoc power analysis based on the observed mean difference in VAS scores at 3 months (mean = 2.0, SD = 1.6). This analysis revealed a power of 85% at an alpha level of 0.05, supporting the adequacy of the sample size in detecting clinically meaningful differences. We acknowledge that multiple comparisons were conducted across time points. However, the primary endpoint was clearly defined a priori, and interpretations were based primarily on this outcome. Correction for multiplicity was not applied due to the exploratory nature of the secondary endpoints. For future research, a mixed-effects model for repeated measures may offer a more robust approach to handling multiple time-point data. Statistical significance was set at *p* < 0.05.

## Results

**Fig. 1:**
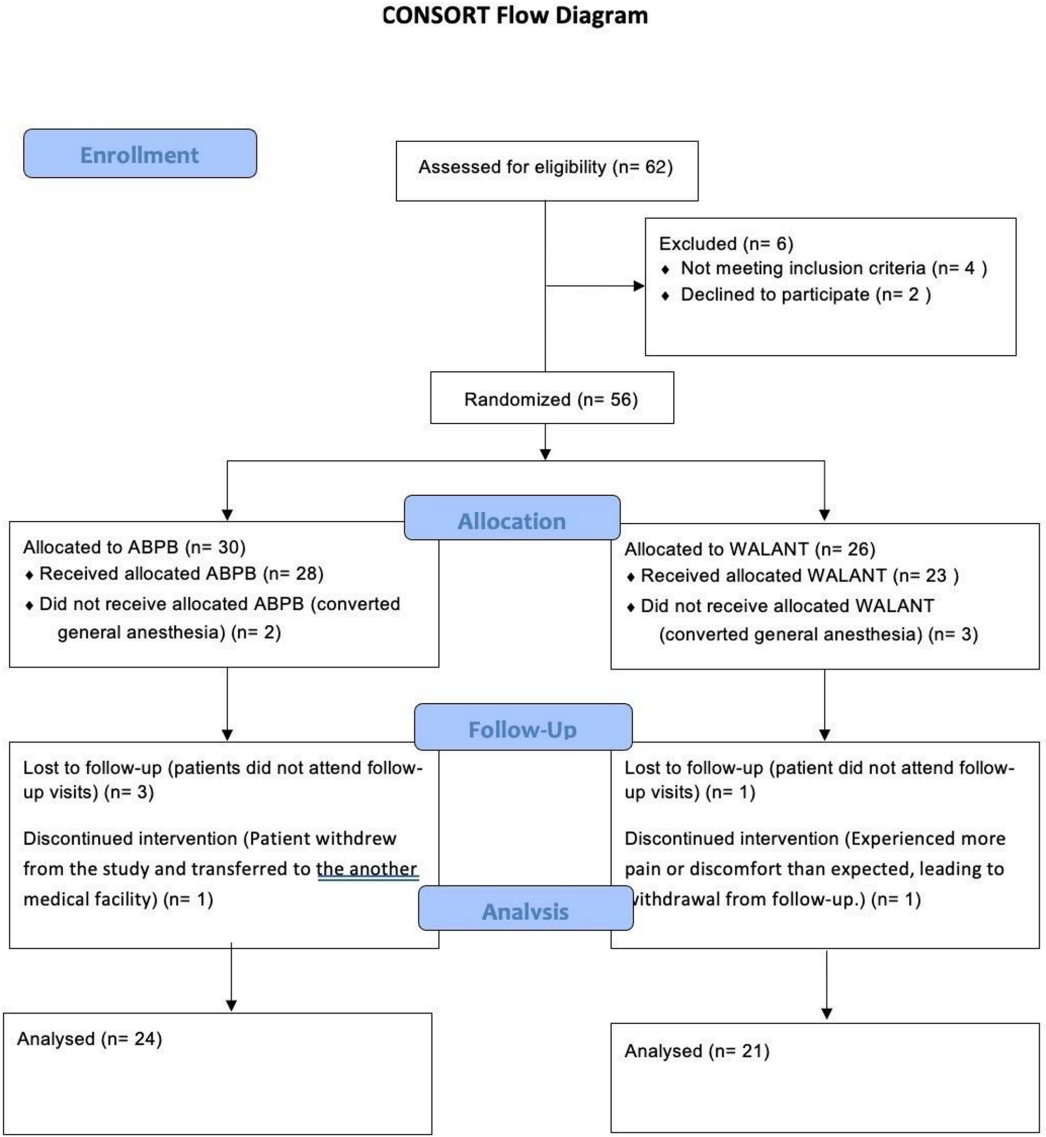
CONSORT flow diagram of patient enrollment, allocation, follow-up, and analysis

A total of 62 patients were assessed for eligibility, of whom 6 were excluded (4 did not meet inclusion criteria, and 2 declined to participate). The remaining 56 patients were quasi-randomly allocated to either the ABPB group (*n* = 30) or the WALANT group (*n* = 26). In the ABPB group, 2 patients were converted to general anesthesia and did not receive the allocated intervention. Additionally, 3 patients were lost to follow-up and 1 patient withdrew after being transferred to another facility, resulting in 24 patients included in the final analysis. In the WALANT group, 3 patients were converted to general anesthesia, 1 was lost to follow-up, and 1 withdrew due to pain or discomfort during the early postoperative period, leaving 21 patients for analysis. The detailed flow of patients is illustrated in Fig. 1. The final cohorts consisted of 24 patients (8 males, 16 females) in the ABPB group and 21 patients (6 males, 15 females) in the WALANT group (Table 1).

**Table 1:** Descriptive evaluation of the patients

	Axillary block (*n* = 24)	WALANT (*n* = 21)	*p*
Age (years)	55.9 ± 11.7	56.1 ± 11.5	0.955*
Time spent in operating room (minute)	41.5 ± 9.4	20.8 ± 4.1	0.001*
VAS score at preop	7.4 ± 1.8	7.1 ± 2.6	0.633^¥^
VAS score at 1 st post-op hour	0.6 ± 2.5	0.07 ± 0.2	0.961*
VAS score at 8th post-op hour	4.8 ± 2.5	3.8 ± 2.5	0.4 ^¥^
VAS score at 1 st post-op day	4.8 ± 2.8	3.1 ± 1.9	0.08^¥^
VAS score at 3rd post-op week	2.5 ± 2.1	0.7 ± 0.8	0.003^¥^
VAS score at 3rd post-op month	2.3 ± 1.4	0.3 ± 0.4	0.003^¥^
USG Thickness (mm)	48.3 ± 4.6	48.9 ± 5.1	0.697^¥^
USG Volume (mm^3^)	70.8 ± 16.03	72.6 ± 11.03	0.718^¥^
Thumb pinch (Newton)	6.2 ± 4.9	9.4 ± 7.4	0.227^¥^
Thumb pinch at 3rd post-op month (Newton)	8.3 ± 4.7	10.67 ± 7.8	0.377*
*p* ^£^	0.001^*£*^	0.05^*£*^	
Pinch other finger (Newton)	41.4 ± 29.2	46.6 ± 30.2	0.611^¥^
Pinch other finger at 3rd post-op month (Newton)	45.5 ± 26.6	49 ± 28.7	0.712^¥^
*p* ^£^	0.05^*£*^	0.736^*£*^	
PSQI at preop	8.2 ± 4.3	7.6 ± 3.7	0.657^¥^
PSQI at 3rd post-op month	5.6 ± 3.6	5.8 ± 2.9	0.868*
*p* ^£^	0.001^*£*^	0.002^*£*^	
BOSTON SSS at preop	35.1 ± 10.3	31.8 ± 9.1	0.466^¥^
BOSTON SSS at 3rd post-op month	22.1 ± 4.6	23.8 ± 6.1	0.505^*¥*^
*p* ^£^	0.002^£^	0.001^£^	
BOSTON FDS at preop	29.1 ± 6.9	28.2 ± 6.9	0.766^¥^
BOSTON FDS at 3rd post-op month	19.4 ± 6.05	22.6 ± 6.4	0.193*
*p* ^£^	0.001^£^	0.001^£^	

* Independent sample t-test, ^¥^ Mann-whitney u test, ^*£*^ *Wilcoson* Signed Rank Test

Independent samples t-test (*) was used for variables with normal distribution; Mann–Whitney U test (¥) was applied to non-normally distributed independent variables. Paired comparisons within groups were analyzed using the Wilcoxon signed-rank test (₤)

*VAS* Visual Analog Scale (0–10 scale), *PSQI* Pittsburgh Sleep Quality Index (Total score, 0–21), *USG Thickness* Transverse Carpal Ligament thickness measured via ultrasonography (mm),* USG Volume* Median nerve cross-sectional volume measured via ultrasonography (mm³), *Thumb pinch* Lateral pinch strength of the thumb (measured in Newtons), *Pinch other finger* Three-jaw chuck pinch strength (measured in Newtons), *BOSTON SSS* Symptom Severity Scale of the Boston Carpal Tunnel Questionnaire (Total score), *BOSTON FDS* Functional Disability Scale of the Boston Carpal Tunnel Questionnaire (Total score)

### Operating room time

Patients in the WALANT group had significantly shorter operative times compared to the ABPB group (20.8 ± 4.1 min vs. 41.5 ± 9.4 min; mean difference: − 20.7 min; 95% CI: − 25.1 to − 16.3; *p* = 0.001) (Table 1).

### Pain assessment

VAS was used to assess somatic pain at various time points (preoperative, postoperative 1 h, 8 h, 1 day, 3 weeks, and 3 months). Pain scores did not significantly differ between groups preoperatively. On the contrary, the WALANT group demonstrated significantly lower pain scores compared to the ABPB group at both postoperative 3 weeks (1.6 ± 1.0 vs. 2.8 ± 1.3; mean difference: − 1.2; 95% CI: − 1.9 to − 0.5; *p* = 0.003) and 3 months (1.1 ± 0.9 vs. 2.4 ± 1.1; mean difference: − 1.3; 95% CI: − 2.0 to − 0.6; *p* = 0.003) (Table 1). Pairwise comparisons of VAS pain scores within groups demonstrated dramatic reductions from preoperative level to one hour postoperatively (mean difference: − 4.8; 95% CI: − 5.7 to − 3.9; *p* = 0.001) (Table 2). Pain scores increased slightly at the 8th postoperative hour but decreased again by the 3rd week. At the 3rd month follow-up, pain scores remained significantly lower in the WALANT group compared to the ABPB group (mean difference: − 1.3; 95% CI: − 2.4 to − 0.1; *p* = 0.034) (Table 2).

**Table 2:** Comparison of pain according to groups

	Axillary block (*p*) ^£^	WALANT (*p*) ^£^
VAS score (preoperative vs. 1 st postoperative hour)	0.001	0.001
VAS score (1st vs. 8th postoperative hour)	0.001	0.001
VAS score (8th postoperative hour vs. 1 st postoperative day)	0.932	0.127
VAS score (1st postoperative day vs. 3rd postoperative week)	0.009	0.002
VAS score (3rd postoperative week vs. 3rd postoperative month)	0.057	0.034

^*£*^ *Wilcoson* Signed Rank Test

£: Wilcoxon Signed Rank Test, within-group comparisons for changes in pain over time. Non-parametric test was preferred due to non-normal distribution of repeated measures

*VAS* Visual Analog Scale (0–10 scale)

### Functional outcomes

No significant between-group differences were observed in grip strength at any time point (*p* > 0.05 for all comparisons). However, both groups demonstrated significant improvements in thumb pinch strength from preoperative to 3-month follow-up (ABPB: *p* = 0.001; WALANT: *p* = 0.050) (Table 1).

### Sleep quality and patient-reported outcomes

There were no statistically significant differences between the groups in PSQI, SSS, or FDS scores at either preoperatively or at the 3-month postoperative time points. However, both groups demonstrated significant change within groups over time from preoperative to 3-month postoperative evaluations in PSQI, SSS, and FDS scores (*p* < 0.05) (Table 1).

### EMG and ultrasound findings

Patients with type 3 EMG findings were older than those with type 2 findings for both groups, as shown by subgroup analysis (ABPB: 60.0 ± 9.98 vs. 46.0 ± 10.11 years; mean difference: 14.0 years; 95% CI: 4.2 to 23.8; *p* = 0.007; WALANT: 58.3 ± 11.9 vs. 47.3 ± 0.57 years; mean difference: 11.0 years; 95% CI: 2.9 to 19.2; *p* = 0.009) (Table 3). Patients with type 2 EMG findings in the WALANT group had significantly thicker transverse carpal ligaments than those with type 3 findings (54.67 ± 3.21 mm vs. 47.5 ± 4.5 mm; mean difference: 7.17 mm; 95% CI: 2.04 to 12.30; *p* = 0.043) on ultrasound evaluation (Table 3).

**Table 3:** Comparison of the results according to EMG

	Axillary block			WALANT		
	EMG 2 (*n* = 10)	EMG 3 (*n* = 14)	p value	EMG 2 (6)	EMG 3 (15)	p value
	Mean ± Std. D	Mean ± Std. D		Mean ± Std. D	Mean ± Std. D	
Age (years)	46 ± 10.11	60 ± 9.98	0.007^*t*^	47.3 ± 0.57	58.33 ± 11.9	0.009 ^*t*^
Thumb pinch (Newton)	8.12 ± 5.35	4.79 ± 3.21	0.098^*¥*^	11.3 ± 5.68	9 ± 7.95	0.64^*¥*^
Pinch other finger (Newton)	54.38 ± 27.4	34.3 ± 23.2	0.09^*¥*^	75 ± 47.7	45.8 ± 23.1	0.13^*¥*^
Thumb pinch at 3rd month (Newton)	10.5 ± 4.7	6.83 ± 3.32	0.057 ^***t***^	12.6 ± 6.65	10.2 ± 8.3	0.64 ^***t***^
Pinch other finger at 3rd month (Newton)	58.75 ± 22.9	38.17 ± 21.6	0.03^*¥*^	73.3 ± 47.2	42.9 ± 21.1	0.1^*¥*^
VAS score at preop	7.38 ± 2.13	6.83 ± 1.6	0.52^*¥*^	8.67 ± 1.52	6.6 ± 2.7	0.25^*¥*^
VAS score at 1 st post-op hour	1.25 ± 3.5	1.2 ± 0.8	0.23 ^***t***^	1.1 ± 0.8	1.1 ± 0.89	0.63 ^***t***^
VAS score at 8th post-op hour	3.8 ± 1.8	5 ± 2.7	0.33^*¥*^	4 ± 2	3.75 ± 2.7	0.88^*¥*^
VAS score at 1 st post-op day	4.13 ± 2.9	5 ± 2.37	0.46^*¥*^	3.3 ± 1.5	3.08 ± 2.15	0.85^*¥*^
VAS score at 3rd post-op week	3.25 ± 2.49	2.25 ± 1.8	0.31^*¥*^	1.33 ± 0.57	0.58 ± 0.9	0.19^*¥*^
VAS score at 3rd post-op month	1.88 ± 1.72	1.75 ± 1.6	0.87^*¥*^	1 ± 0.0	0.17 ± 0.38	0.008^*¥*^
USG Thickness (mm)	47.75 ± 5.8	47 ± 4.57	0.75^*¥*^	54.67 ± 3.21	47.5 ± 4.5	0.043^*¥*^
USG Volume (mm^3^)	71.5 ± 13.5	65.17 ± 19.14	0.43^*¥*^	80.3 ± 4.1	70.6 ± 11.4	0.18^*¥*^
PSQI at preop	9.25 ± 5.89	8.5 ± 3.5	0.72^*¥*^	6.3 ± 4.6	8 ± 3.6	0.5^*¥*^
PSQI at 3rd post-op month	7.25 ± 4.7	5.4 ± 2.8	0.29 ^***t***^	4.3 ± 3.05	6.2 ± 2.9	0.3 ^***t***^
BOSTON SSS preop	39.75 ± 9.2	30.5 ± 9	0.03^*¥*^	33.3 ± 9.6	31.5 ± 9.2	0.76^*¥*^
BOSTON FDS preop	28.8 ± 8.4	30.25 ± 7.6	0.7^*¥*^	22.67 ± 8.5	29.58 ± 7.5	0.18^*¥*^
BOSTON SSS at 3rd post-op month	23.63 ± 4.95	20.83 ± 4.68	0.21^*¥*^	20.6 ± 3.05	24.5 ± 6.4	0.33^*¥*^
BOSTON FDS at 3rd post-op month	19.9 ± 5.4	20.1 ± 6.8	0.94 ^***t***^	17.3 ± 5.5	24 ± 6.1	0.11 ^***t***^

^*t*^ Independent sample t-test, ^*¥*^ Mann-whitney u test

*t* Independent sample t-test for parametric data, *¥* Mann-Whitney U test for non-parametric data between EMG 2 and EMG 3 groups

*VAS* Visual Analog Scale (0–10 scale), *PSQI* Pittsburgh Sleep Quality Index (Total score, 0–21), *USG Thickness* Transverse Carpal Ligament thickness measured via ultrasonography (mm), *USG Volume* Median nerve cross-sectional volume measured via ultrasonography (mm³), *Thumb pinch* Lateral pinch strength of the thumb (measured in Newtons), *Pinch other finger* Three-jaw chuck pinch strength (measured in Newtons), *BOSTON SSS* Symptom Severity Scale of the Boston Carpal Tunnel Questionnaire (Total score), *BOSTON FDS* Functional Disability Scale of the Boston Carpal Tunnel Questionnaire (Total score)

## Discussion

This study addresses a significant gap in existing research through presenting the first investigation into the correlation between the type of anaesthesia utilised in CTR and grip strength as well as sleep quality. The study primarily aimed to compare postoperative pain at 3 months between the two anesthesia techniques. Secondary outcomes included grip strength and sleep quality at the same time point. This prospective observational study with quasi-randomization found that patients in the WALANT group reported lower pain ratings at both three weeks and three months postoperatively compared to those in the ABPB group. Additionally, the WALANT technique was associated with shorter operating times. Functional recovery, as measured by grip strength, was similar between groups, indicating that anaesthetic type may not influence motor recovery. Similarly, both groups showed comparable improvements in sleep quality, suggesting that postoperative sleep disturbances are likely influenced by factors other than anaesthesia type.

Multiple investigations have quantified the time efficiency achieved when CTR is conducted under WALANT as in line with the current study. However, previous studies have often included the post-anesthesia care unit (PACU) duration in their assessments, whereas our study specifically focused on operative time, defined as the period from operating room entry to exit, excluding PACU stay. For instance, Alter et al. [13] and Via et al. [14] reported significant time efficiencies in WALANT patients, mainly due to reduced PACU stay, with average time savings of 77 and 22 min, respectively. Similarly, Kamal et al. [15] developed a streamlined workflow for WALANT, incorporating preoperative anesthesia administration and immediate postoperative care, leading to increased overall efficiency. In line with our findings, Soydan et al. [16] reported a mean operative time of 11 min (range, 8–18 min) for WALANT procedures and Aykaç et al. [17] reported a mean operative time of 16.75 ± 2.39 min for non-tourniquet WALANT procedures. These studies, which specifically assessed operative time without considering PACU duration, further support our results. Our findings suggest that the efficiency of WALANT extends beyond PACU-related factors and is also evident within the intraoperative period. The reduction in operative time may be attributed to minimized transition delays, faster patient positioning, and improved workflow optimization. It is important to note that our operative time definition (entry to exit of the operating room) inherently includes anesthesia preparation time, which tends to be longer in ABPB cases due to the time needed for nerve block administration and confirmation. This may have contributed to the longer total operative time observed in the ABPB group. Although ABPB offers robust analgesia, its application in the operating room setting may prolong total operative room time due to the time needed for ultrasound guidance, drug preparation, and injection. Strategies such as synchronizing block performance with surgical team preparations, using standardized block trays, and enhancing inter-team communication may help reduce delays and optimize workflow efficiency.

The current study showed that WALANT provides enhanced pain reduction at 3 weeks and 3 months following surgery, exhibiting substantially lower VAS assessments in comparison to ABPB. Consistent with earlier research on WALANT’s effectiveness in CTR, these results also reveal some unique benefits in the intermediate term. Several studies have shown that WALANT offers good early pain relief following surgery because of the lack of tourniquet-induced ischaemic pain [18]. However, we found that this benefit continues for at least three months after surgery, which has not been extensively studied before. Faraz et al. [18] did not evaluate pain outcomes at subsequent time periods; they only reported that WALANT patients had substantially decreased VAS pain levels after 2 weeks. In contrast, Okamura et al. [19] compared WALANT with intravenous regional anestesia (IVRA) in a randomised clinical study; they discovered that WALANT provided better pain relief in the beginning, but there was no significant difference in VAS scores at 3 months. In addition, Tulipan et al. [20] discovered that although initial pain levels were reduced in the MAC group, there was no statistically significant change in pain scores at the three-month mark. One probable reason might be that WALANT makes early mobilisation more achievable, which in turn reduces the likelihood of perineural adhesions, improves functional recovery in the long term, and, finally, leads to less pain perception. This variance may arise from variations in patient selection, surgical methods, or rehabilitation regimens, indicating the necessity for additional exploration into the long-term analgesic benefits of WALANT. Furthermore, potential physiological mechanisms underlying the observed benefits of WALANT may include reduced ischemia–reperfusion injury by eliminating tourniquet use, thus avoiding acute occlusion and reperfusion-associated tissue stress. Supporting this, studies indicate tourniquet use is associated with increased postoperative pain and swelling due to ischemic insult in musculoskeletal tissues [21]. Additionally, WALANT maintains stable tissue perfusion throughout surgery, which may help modulate local nociceptor activity and reduce inflammatory pain — consistent with clinical observations of lower postoperative pain with WALANT in hand surgery procedures [22]. Furthermore, the difference in postoperative pain trajectories between groups may also be influenced by the distinct anesthetic regimens used. In our study, the WALANT group received 1% lidocaine with epinephrine (1:100,000) and sodium bicarbonate, consistent with the WALANT consortium protocol and standard clinical practice. In contrast, the ABPB group received a mixture of lidocaine and bupivacaine, which is a commonly accepted regimen for longer-acting peripheral nerve blocks. While bupivacaine has a longer duration of action in the immediate postoperative phase, our findings indicate that the WALANT technique provided superior analgesia at 3 weeks and 3 months postoperatively. This paradox may be explained by improved early mobilization and reduced perineural inflammation, potentially attributable to the absence of tourniquet-related ischemic insult in the WALANT group. These findings highlight that long-term analgesic outcomes are not solely determined by the pharmacological duration of anesthetic agents, but also by perioperative surgical and rehabilitation dynamics.

While a notable difference in pain scores was identified between the groups, this disparity was not evident in the Boston SSS. This discrepancy could stem from the comprehensive approach of the Boston SSS, which assesses various symptoms including numbness, tingling, and strength, rather than concentrating exclusively on pain. Furthermore, our study lacked sufficient power for subgroup analysis, and the limited sample size may have constrained our ability to identify differences in specific components of the Boston SSS. Further studies involving larger cohorts and focused sub-analyses could enhance our understanding of these findings.

The current study appears to be the sole inquiry in the literature that compares grip strength and quality of sleep between the WALANT and ABPB groups in patients undergoing CTR. Our results show that the WALANT and ABPB groups did not vary significantly in terms of grip strength, indicating that although WALANT performs a better job of reducing postoperative pain, it does not directly accelerate the recovery of grip strength. Tulipan et al. [20] also compared the WALANT and MAC groups’ DASH and Levine-Katz scores, which are similar to the SSS and FDS scores used in our study. They found no statistically significant differences in clinical outcomes at 2 weeks or 3 months post-surgery, which is in line with our results. Nonetheless, the aforementioned study did not assess sleep quality and grip strength. There are three recognised parameters that influence the recovery of grip strength after CTR. These include TCL function [23], pillar pain [24], and decompressed median nerve healing [25, 26]. Several studies indicate that grip strength recovery is generally reduced in patients with diabetes relative to those without the condition. This may result from insufficient nerve recovery in individuals with diabetes [25, 26]. Compared to open CTR, endoscopic CTR reduced grip strength and sped up healing in the initial weeks after surgery for pillar pain, but there was no significant change in grip strength following long-term follow-up [24]. In regards to the TCL, a study conducted by Netscher et al. [27] contrasted CTR with and without reconstructing of the TCL. The results showed that both groups regained their preoperative grip strength by 12 weeks, although the group that had reconstruction showed a faster recovery. This study stands out because it is the first to examine the impact of grip strength and anaesthetic type, which none of the others have done. Lee et al. [28] indicated that grip strength often recovers to at least preoperative levels within 3 to 6 months post-surgery, and that recovery may differ in the short term based on the surgical technique employed rather than the kind of anaesthesia used, consistent with the findings of the current study. They asserted that there is no substantial difference in long-term follow-up.

Enhancing sleep quality is a crucial objective in the treatment of individuals with CTS, as sleep disruptions significantly affect a patient’s quality of life. Approximately 80% of patients with CTS exhibited clinically significant sleep disturbances, indicated by a PSQI score exceeding 5 [29]. Several studies have demonstrated the positive impact of the CTD on sleep disorders. This outcome is regarded as a significant benefit of the surgical treatment for patients with CTS, as enhancements in sleep quality contribute to overall patient satisfaction and quality of life. In alignment with our findings, Niedermeier et al. [30] observed an enhancement in sleep disorders as assessed by the PSQI, with a mean score decreasing from 10.4 points preoperatively to 7.8 points at two weeks post-surgery and further to 6.4 points at six weeks post-surgery. Yalın et al. [31] demonstrated that enhancements in sleep quality following CTD persisted for up to 6 months in both young and elderly individuals. A prospective research study by Tulipan et al. [32] found that CTD improved sleep quality within 7 days. The authors noticed no significant change in sleep quality between 2 weeks and 3 months. In this prospective study, we observed an improvement in sleep quality for up to 3 months in all patients, regardless of the type of anaesthesia used. The current prospective study addresses the limited research available regarding the impact of anaesthesia type on postoperative sleep quality [33]. It found no significant difference between WALANT and ABPB anaesthesia concerning postoperative sleep quality in CTS patients, thereby contributing valuable insights to the existing literature on this topic.

### Strengths and limitations

This research possesses several strengths. The prospective approach effectively reduces recall bias and provides that patients’ experiences are documented in real time, rather than depending on retrospective perceptions that may be affected by altering perspectives over time. Furthermore, no prior research has ever compared WALANT with ABPB for CTR patients’ mid-term pain alleviation, grip strength, or sleep quality. The current study is even more rigorous in its methodology since it uses standardised surgical methods and objective functional assessments. Nevertheless, it is important to acknowledge a few restrictions. To begin, our results may not be applicable to a broader population due to the limited sample size. One limitation of this study is the lack of a priori sample size calculation. Although a post hoc power analysis suggested adequate statistical power for the primary outcome, future prospective studies should incorporate an a priori power analysis during the study design phase. Additionally, multiple comparisons were made without correction for multiplicity, which may increase the risk of type I error. A mixed-model analysis could have provided a more robust statistical framework for repeated measures. Secondly, while pain, strength of grip, and quality of sleep were evaluated at 3 months postoperatively, this follow-up period may be insufficient to detect long-term functional differences and potential late-emerging effects of anaesthesia on recovery. Extending the follow-up duration would be advantageous to capture a more comprehensive trajectory of outcomes. Thirdly, while PSQI was used to measure sleep quality, other factors including sleep fragmentation, latency as well as polysomnographic data were not taken into account, which hindered an overall better comprehension of sleep recovery. Additionally, certain potentially relevant confounders such as patients’ work status, hand dominance, and pre-existing sleep disturbances were not systematically collected or controlled for in our analysis. Other unmeasured variables such as concurrent medication use (e.g., sedatives or analgesics), baseline sleep habits, or pain catastrophizing tendencies could also confound subjective outcomes. This may have introduced residual confounding, particularly for subjective outcomes such as postoperative pain perception and sleep quality. Fourthly, although the WALANT and ABPB methods were standardised, there may have been unanticipated confounding effects due to differences in surgical expertise and postoperative rehabilitation programs. In addition, staff-related factors (e.g., nursing care variability, anesthesiologist preferences), day-of-week scheduling effects, and individual surgeon operating time experience were not fully accounted for, potentially influencing recovery trajectories or operative efficiency. Fifthly, although allocation by calendar days was used to avoid patient preference and logistical difficulties, this method does not constitute true randomization. Its predictable nature may have introduced selection bias, thereby compromising internal validity, particularly in a study assessing subjective outcomes. Additionally, early pain outcomes were not blinded due to the nature of the interventions, introducing a potential risk of detection and performance bias. This limitation should be considered when interpreting short-term VAS results. Future studies should adopt computer-generated or blocked randomization methods to ensure unbiased group allocation and improve methodological rigor. Furthermore, the current study was performed at a single centre, thus constraining its external validity, as variations in surgical procedures, healthcare systems, and recovery protocols among institutions might affect results. Although we standardized operative time as “entry-to-exit” in the OR to ensure fairness, minor variations in team efficiency or room turnover logistics may still influence total duration. Additionally, full blinding of patients and outcome assessors was not feasible due to the nature of the anesthesia techniques. Despite employing independent assessors, the risk of performance and detection bias remains, particularly for subjective outcomes such as pain and sleep quality. Also, this study followed a per-protocol analysis approach. Patients who were lost to follow-up or did not receive the allocated intervention (e.g., conversion to general anesthesia) were excluded from the final analysis. Therefore, an intention-to-treat (ITT) analysis was not performed, which may introduce selection bias and limit the generalizability of the findings. Furthermore, tourniquet use was not applied in WALANT cases, while all ABPB patients underwent tourniquet placement, introducing a systematic difference that may impact pain scores. The influence of this variable on postoperative discomfort or ischemia-reperfusion-related inflammation should be acknowledged. Finally, psychosocial factors, including depression, anxiety, and baseline patient expectations, which are recognised to influence the quality of sleep and postoperative pain perception, were not systematically assessed, potentially affecting the interpretation of sleep outcomes.

## Conclusion

This prospective observational study with quasi-randomization provides insights into the mid-term outcomes of WALANT and ABPB anaesthesia in CTR. Our findings suggest that WALANT is a feasible anaesthetic option for CTR, with shorter operative time and lower postoperative pain scores at three months. Although both procedures resulted in similar functional recovery, grip strength, and quality of sleep enhancements, WALANT’s reduced operational duration and enhanced pain management underscore its therapeutic advantages. Our findings indicate that postoperative sleep recovery is affected by factors beyond mere pain management, highlighting the complex nature of sleep disruptions in CTS patients. Furthermore, both groups exhibited comparable recovery in grip strength, supporting the notion that the kind of anaesthesia does not directly affect the restoration of motor function after CTR. Further study ought to concentrate on longitudinal studies including larger cohorts to better investigate the influence of anaesthesia selection on functional and quality of life indicators in CTR patients.

## Data Availability

The datasets generated during and/or analyzed during the current study are available from the corresponding author on reasonable request.
